# Progressive increase of cardiometabolic risk in Brazilian children according to obesity phenotypes

**DOI:** 10.1038/s41430-026-01700-x

**Published:** 2026-02-05

**Authors:** Bruna Clemente Cota, Mariana de Santis Filgueiras, Nalva de Paula Dias, Leidjaira Lopes Juvanhol, Patrícia Feliciano Pereira, Juliana Farias de Novaes

**Affiliations:** 1 Department of Nutrition and Health, Universidade Federal de Viçosa (UFV), Av. P.H. Rolfs s/n, Campus Universitário, Viçosa, Minas Gerais Brazil; 2 Instituto de Saúde Coletiva, Universidade Federal do Estado do Rio de Janeiro (Unirio), Rio de Janeiro, RJ Brazil

**Keywords:** Biomarkers, Obesity

## Abstract

**Objective:**

We investigated the association of obesity phenotypes with MetS and components scores, subclinical inflammation, anti- and oxidative markers in children.

**Subjects/Methods:**

This is a cross-sectional study with 364 children (8 and 9-year-olds) in Viçosa, Minas Gerais, Brazil. Children were classified as: 1.“normal-weight lean” (NWL) when they had normal-weight by BMI and adequate fat percentage assessed by DXA; 2. “normal-weight obesity” (NWO) for those with normal-weight and excess fat; and 3. “excess weight” for those with overweight/obesity and excess fat. The score for the MetS and its components was estimated, and the inflammatory and oxidative stress markers were measured. Multiple linear regression was used.

**Results:**

Of thirteen cardiometabolic risk factors investigated, five were positively associated with the NWO, compared to NWL. Moreover, eleven cardiometabolic risk factors were associated with excess weight, compared to NWL. When the two phenotypes of excess body fat were contrasted, we found eight cardiometabolic risk factors associated with excess weight, compared to NWO.

**Conclusion:**

An intermediate cardiometabolic risk was observed in children with the NWO phenotype when comparing the status of NWL to excess weight. This study reinforces the importance of investigating cardiometabolic risk in early ages, independent of BMI.

## Introduction

The rapid increase in obesity prevalence in childhood is considered a worldwide concern [1], since it is associated with the incidence of several chronic diseases in adulthood [2], generating a negative impact on public health [3]. Evidence has shown that obesity is related to components of metabolic syndrome (MetS), subclinical inflammation, and oxidative stress in children [4, 5].

The higher adipose tissue mass can lead to impaired secretion of adipokines, such as leptin and adiponectin [6, 7], oxidative status [8], and the progression of chronic diseases, including insulin resistance and chronic inflammation [9, 10]. Furthermore, elevated serum uric acid (SUA)—a metabolite of purine degradation—has been associated with obesity and metabolic disorders in children and adolescents [11, 12]. At high concentrations, SUA assumes pro-oxidant activity, which is related to increased enzymatic activity of xanthine oxidase, excessive production of reactive oxygen species and pro-inflammatory cytokines, which can propagate chain reactions and cause biological damage [13–15].

The body mass index (BMI) has been used for the obesity diagnosis. However, it presents limitations since it does not differentiate the body tissues and may underestimate or overestimate adiposity, which can hinder clinical approaches to health care and policy [16]. In this context, people with normal weight may have excess body fat and/or metabolic disorders [17], which has been termed the normal-weight obesity (NWO) phenotype. Previous studies have indicated that NWO is related to increased blood glucose, diabetes, high triglycerides (TG), and low high-density lipoprotein (HDL) [18, 19]. However, most studies were carried out in adults, and we did not identify studies that evaluated the relationship of inflammatory and oxidative stress markers with NWO in children.

Considering the increase in childhood adiposity and the limitations of BMI for an accurate diagnosis of obesity, it is urgent to investigate cardiometabolic risk factors in children with NWO phenotype, as they should be included in the screening process to improve the health conditions, despite normal BMI. The aim of this study was to investigate the association of obesity phenotypes with MetS score and its components, subclinical inflammation, anti- and oxidative markers in children. Our hypothesis is that children with the NWO phenotype have an intermediate cardiometabolic risk, compared to the status of normal-weight lean (NWL) to excess weight.

## Methods

### Participants and study design

This is a cross-sectional study as part of the “Schoolchildren Health Assessment Survey” (PASE, in Portuguese), which enrolled a representative sample of children aged 8 and 9 years, from all public and private schools in the urban area of Viçosa, Minas Gerais, Brazil.

Sample size was calculated using Epi Info software (version 7.2; Atlanta, GA), from a specific formula for cross-sectional studies. We considered the total number of schoolchildren in Viçosa aged 8 and 9 years (*n* = 1464) in 2015 [20], expected prevalence of 50% [21], tolerated error of 5%, and 95% confidence level for the calculation of sample size (*n* = 305). We added 15% for losses, resulting in a minimum sample of 351 children.

The schoolchildren were selected by stratified random sampling. The sample from each school met the proportionality ratio of students enrolled by age and sex. The selection of children was done by a random simple draw until the necessary number for each school was completed.

Non-inclusion criteria were considered as health problems that affected the children’s nutritional status or body composition, chronic use of medication that influenced glucose and/or lipid metabolism, or failure to contact primary care providers after three attempts.

A pilot study was made with 37 children aged 8–9 years enrolled in a school selected randomly. These children did not participate in the final sample. The pilot study was carried out to test the questionnaires.

This study was conducted according to the guidelines established in the Declaration of Helsinki and approved by the Ethics Committee on Human Research of the *Universidade Federal de Viçosa* (UFV) (process no. 663.171/2014). All parents or guardians of children signed the Informed Consent Form.

### Biochemical assays

In a 12-h fast, the blood samples were collected by venipuncture in the antecubital region at the UFV Health Center’s clinical analysis sector. The biochemical components of MetS (HDL-c, TG, fasting glucose, and insulin), markers of inflammation (high-sensitivity C-reactive protein (hs-CRP) and adipokines (leptin and adiponectin), and pro- (malondialdehyde—MDA and SUA) and antioxidants (superoxide dismutase—SOD and ferric reducing antioxidant power—FRAP) were evaluated.

HDL-c, TG, fasting glucose, uric acid, and hs-CRP were measured in serum using automatic equipment (BioSystems 200 Mindray® model, Nanchan, China), according to the recommendations of the manufacturer of the Bioclin® kit used (Belo Horizonte, MG, Brazil).

Fasting insulin was determined by the chemiluminescence immunoassay method and quantified by the Elecsys Insulin® test (Roche Diagnostics, Indianapolis, IN, USA). The homeostatic model assessment for insulin resistance (HOMA-IR) was calculated according to the equation: fasting insulin (μU/mL) × fasting blood glucose (mmol/L)/22.5 [22].

Serum leptin was measured by the enzyme immunoassay method, with a coefficient of variation intra-assay <13.3% and inter-assay <12.7% (KAP2281, DIAsource®, Louvain-la-Neuve, Belgium). Plasma adiponectin was determined by commercial ELISA sandwich kits, with coefficients of variation intra-assay <10% and inter-assay <12% (human adiponectin: SEA605Hu, Cloud Clone Corp.®, Houston, TX, USA). Adipokines were analyzed in duplicate.

For the determination of oxidative residues, the measures were made in triplicate in plasma. SOD dosage was based on the rate of pyrogallol autoxidation (ACS reagent, 254002, Sigma-Aldrich, St. Louis, MO, USA); MDA in the measurement of thiobarbituric acid reactive species (TBARS); and FRAP was based on the ability of antioxidants to reduce ferric iron (Fe3 + ). The analysis was performed in polystyrene microplates using the FRAP solution. Trolox solution (97%, 238813, Sigma-Aldrich) was used as an antioxidant agent to determine the standard curve and the reading was performed on a Thermo Scientific Multiskan GO Microplate Spectrophotometer at a wavelength of 570 nm (SOD), 535 nm (MDA), 594 nm (FRAP). Analyzes were obtained on the standard curve.

### Blood pressure

Blood pressure was measured three times using digital blood pressure monitors (Omron®, Vernon Hills, IL, USA), with the child in a sitting position after resting for at least 5 min. The mean arterial pressure (MAP) was calculated [MAP = 1/3(SBP) + 2/3(DBP)] from systolic (SBP) and diastolic (DBP) blood pressures’ mean, in mmHg [23].

### MetS and components scores

There is no clear definition of MetS in children, and a continuous MetS score has been estimated using criteria similar to those for adults [24, 25]. It was recommended that the five main variables of MetS be used to calculate the score in research, including (1) central obesity (measured by waist circumference—or BMI and/or skinfold thickness if waist circumference is not available), (2) low HDL-c, (3) elevated triglycerides, (4) elevated blood pressure (systolic and/or diastolic and/or MAP), and (5) abnormal glucose metabolism (impaired fasting glucose, impaired glucose tolerance, and/or HOMA-IR) [24]. In this study, waist circumference (WC) was used to assess central obesity and was measured with the use of an inelastic measuring tape, midway between the inferior margin of the ribs and the superior border of the iliac crest [26]. MAP was used as a criterion for assessing arterial pressure, and HOMA-IR as a measure of insulin resistance [25].

We calculated Z scores for WC, serum TG and HDL-c, HOMA-IR, and MAP by regressing each log-transformed component on sex and log-transformed age in linear regression models to obtain standardized residuals. After the HDL-c score was multiplied by -1, the overall score was calculated as the average of the five component scores. Higher values correspond to a worse metabolic profile [24, 25]. We also evaluate the standardized residuals for each of the five MetS components.

### Normal-weight obesity (NWO) phenotype

NWO phenotype was identified when the child had a normal weight, according to BMI-for-age, and high body fat, simultaneously [27].

BMI-for-age z score was calculated by weight (kilograms) divided by the square of height (meters), according to sex, for the classification of nutritional status through the WHO AnthroPlus software [28]. Body fat (%) was assessed using the Dual Energy X-ray Absorptiometry (DXA) method (Lunar Prodigy Advance, GE Medical Systems Lunar, Milwaukee, WI, USA). The high percentage of body fat was defined as values equal to or greater than 25% and 20% for girls and boys, respectively [29].

In this study, children with: 1. thinness (*n* = 12) were excluded; 2. normal-weight and adequate body fat percentage (*n* = 176) were classified as NWL; 3. normal-weight and high body fat (*n* = 66) were classified as NWO phenotype; and 4. overweight (*n* = 65) and obesity (*n* = 59) were grouped into the excess weight group. Considering the limitations of BMI in differentiating body tissues, two children with excess weight and adequate body fat percentage were excluded from the analyses in the group with excess weight, since all children in this group presented excessive body fat percentage.

### Covariates

Information on the child’s age (years), sex (female and male), screen time (hours/day), and per capita family income (USD) was filled out in a semi-structured questionnaire during an interview with the child and their guardian. Screen time (spent on screen activities such as video games, computer, television, cell phone, or tablet) higher than 2 hours/day was defined as sedentary behavior [30]. Caloric intake was assessed using the average number of calories ingested from three 24-h recalls administered to the study volunteers.

### Data analysis

#### Exposure

Obesity phenotypes (NWL, NWO, and excess weight).

#### Outcomes

MetS and components scores (MetS overall, WC, MAP, HOMA-IR, TG, and HDL-c in Z score), anti- and inflammatory markers (adiponectin, CRP, and leptin), anti- and oxidative status (FRAP, SOD, MDA and SUA).

#### Covariates

Sex, age, per capita income, screen time, and caloric intake.

#### Statistical analyses

The consistency and distribution of numerical variables were evaluated by histograms, and skewness and kurtosis coefficients; the Shapiro-Wilk was used for the normality test. Categorical variables were expressed as absolute and relative frequencies; numerical variables were expressed as mean and standard deviation (SD) or median and interquartile range (IR). Statistical differences for numerical variables according to the categories of obesity phenotypes were analyzed by one-way analysis of variance (ANOVA) (with Bonferroni post-hoc test) or Kruskal-Wallis (with Dunn post-hoc test). Statistical differences for categorical variables were calculated by the chi-square test for trend.

Multiple linear regression with robust estimates of the variance, which are consistent to heteroskedasticity and non-normality [31], were performed to evaluate the associations of the obesity phenotypes with MetS and its components scores, inflammatory markers, and anti- and oxidative markers. Independent models for each outcome variable were estimated. Coefficients (β) with 95% confidence intervals (CI) were calculated, adjusted by sex, age, per capita income, screen time and caloric intake. The adjustment variables were defined by theoretical and statistical criteria [32]. In addition, the p for trend was calculated. All analyses were performed in the Stata® version 14 (StataCorp LP, College Station, TX, USA). The statistical significance level was set to 5%.

## Results

From a sample of 364 children, 52.5% (*n* = 191) were girls, 18.1% (*n* = 66) had NWO phenotype, and 33.5% (*n* = 122) had excess weight. Among normal-weight children (*n* = 242), a relevant percentage presented NWO (27.3%). We observed that per capita income increased progressively according to phenotypes (NWL, NWO and excess weight, respectively), and that mean daily caloric intake was higher in the NWO group compared to NWL group (*p* < 0.05) (Table 1).

**Table 1:** Characterization of the sample of children in Viçosa, Minas Gerais, Brazil, 2015.

	Total sample (*n* = 364)		NWL (*n* = 176)		NWO (*n* = 66)		Excess weight (*n* = 122)		
	Mean or Median or *n*	SD or IR or %	Mean or Median or *n*	SD or IR or %	Mean or Median or *n*	SD or IR or %	Mean or Median or n	SD or IR or %	*p*-value
**Sex**									
Female	191	52.5	89	50.6	36	54.6	66	54.1	0.50^*^
Male	173	47.5	87	49.4	30	45. 5	56	45.9	
**Age (years)**^**1**^	8.5	0.5	8.5	0.5	8.5	0.5	8.6	0.5	0.57
**Per capita income (USD)**^**2**^	149.6	93.7;240.2	120.1^a^	79.6;200.1	156.7^b^	120.1;329.1	153.9^c^	95.3;284.5	**<0.001**
**Screen time**									
≤2 h	189	51.9	97	55.1	32	48.5	60	49. 2	0.25^*^
>2 h	175	48.1	79	44. 9	34	51.5	62	50. 8	
**Caloric intake**^**1**^	1405.5	450.2	1333.6^a^	406.4	1525.7^b^	459.8	1444.2^a,b^	488.5	**0.006**

*NWL* normal-weight lean. *NWO* normal-weight obesity. *SD* standard deviation. *IR* interquartile range.

Data are presented as absolute and relative (%) frequencies; ^1^ Mean (standard deviation); or ^2^ Median (interquartile range).

*Linear trend chi-square.

¹ANOVA (with Bonferroni post-hoc test); or ² Kruskal-Wallis (with Dunn post-hoc test). Different letters indicate statistical difference.

Values in bold (*p* < 0.05).

We found a progressive increase in MetS overall, WC, and HOMA-IR scores, and SUA, according to phenotypes (NWL, NWO and excess weight, respectively) (*p* < 0.001). Furthermore, children with excess weight had higher MAP, TG scores, CRP, and FRAP, compared to NWL and NWO children (*p* < 0.05 in the post-hoc test). Lower HDL-c score, MDA and adiponectin were found in the excess weight group compared to the group with NWL phenotype (*p* < 0.05 in the post-hoc test). Furthermore, we observed a higher leptin value in children with excess weight, when compared to those with NWL and NWO phenotype; and a lower value in those with NWO compared to those with NWL (*p* < 0.05 in the post-hoc test) (Table 2).

**Table 2:** Metabolic syndrome (MetS) and components scores, markers of inflammation, and anti- and oxidative status according to obesity phenotypes in children.

	Total sample (*n* = 364)		NWL (*n* = 176)		NWO (*n* = 66)		Excess weight (*n* = 122)		
	Mean or Median	SD or IR	Mean or Median	SD or IR	Mean or Median	SD or IR	Mean or Median	SD or IR	***p*-value**
***MetS and components scores***									
MetS overall (Z score)	−0.03	−0.44; 0.37	−0.32^a^	−0.64; −0.03	−0.06^b^	−0.39; 0.30	0.55^c^	0.24; 0.98	**<0.001**
Waist circumference (Z score)	−0.23	−0.71; 0.71	−0.71^a^	−0.99; −0.41	−0.17^b^	−0.35; 0.16	1.04^c^	0.69; 1.56	**<0.001**
Mean arterial pressure (Z score)^1^	0.01	1.00	−0.32^a^	0.95	−0.15^a^	0.82	0.59^b^	0.89	**<0.001**
HOMA-IR (Z score)^1^	0.0003	0.98	−0.40^a^	0.85	−0.001^b^	0.91	0.57^c^	0.90	**<0.001**
Triglycerides (Z score)	−0.02	−0.70; 0.55	−0.22^a^	−0.87; 0.39	−0.09^a^	−0.70; 0.46	0.34^b^	−0.38; 1.06	**<0.001**
HDL-c (Z score)^1^	0.004	1.01	0.10^a^	1.03	0.09^a,b^	0.99	−0.20^b^	0.96	**0.03**
***Inflammatory markers***									
CRP (mg/L)	0.001	0.001; 0.96	0.001^a^	0.001; 0.29	0.001^a^	0.001; 0.73	0.23^b^	0.001; 1.74	**<0.001**
Leptin (ng/mL)	1.80	0.40; 6.70	1.15^a^	0.10; 2.60	0.90^b^	0.30; 5.40	9.55^c^	2.00; 18.90	**<0.001**
Adiponectin (µg/mL)	11.31	8.09; 16.74	12.33^a^	8.24; 17.32	11.43^a,b^	8.27; 17.08	10.52^b^	7.93; 15.35	0.16
***Anti- and oxidative markers***									
FRAP (U/mL)	73.54	61.46; 86.94	66.91^a^	57.05; 84.25	75.28^a^	64.10; 85.90	77.74^b^	66.64; 92.00	**<0.001**
SOD (U/mL)	65.68	48.95; 90.91	64.52^a^	48.24; 88.83	78.35^a^	51.78; 93.17	63.88^a^	49.11; 92.80	0.42
MDA (µM/L)	6.44	5.00; 7.81	6.51^a^	5.24; 8.11	6.40^a,b^	4.65; 7.59	6.39^b^	4.91; 7.49	0.15
Serum uric acid (mg/dL)	3.10	2.6; 3.70	2.80^a^	2.40; 3.40	3.20^b^	2.80; 3.60	3.55^c^	3.10; 4.10	**<0.001**

*NWL* normal-weight lean. *NWO* normal-weight obesity. *SD* standard deviation. *IR* interquartile range. *HOMA-IR* homeostasis model assessment for insulin resistance. *HDL-c* high-density lipoprotein cholesterol. *CRP* C-reactive Protein. *FRAP* ferric reducing antioxidant power. *MDA* malondialdehyde. *SOD* superoxide dismutase.

Median (interquartile range - IR); ^1^ Mean (standard deviation-SD).

Kruskal-Wallis (with Dunn post-hoc test), or ^1^ ANOVA (with Bonferroni post-hoc test). Different letters indicate statistical difference.

Values in bold (*p* < 0.05).

Viçosa, Minas Gerais, Brazil, 2015.

### MetS and components scores, inflammatory markers, and anti- and oxidative markers in regression analysis

#### Normal-weight (NWO vs. NWL)

**Fig. 1:**
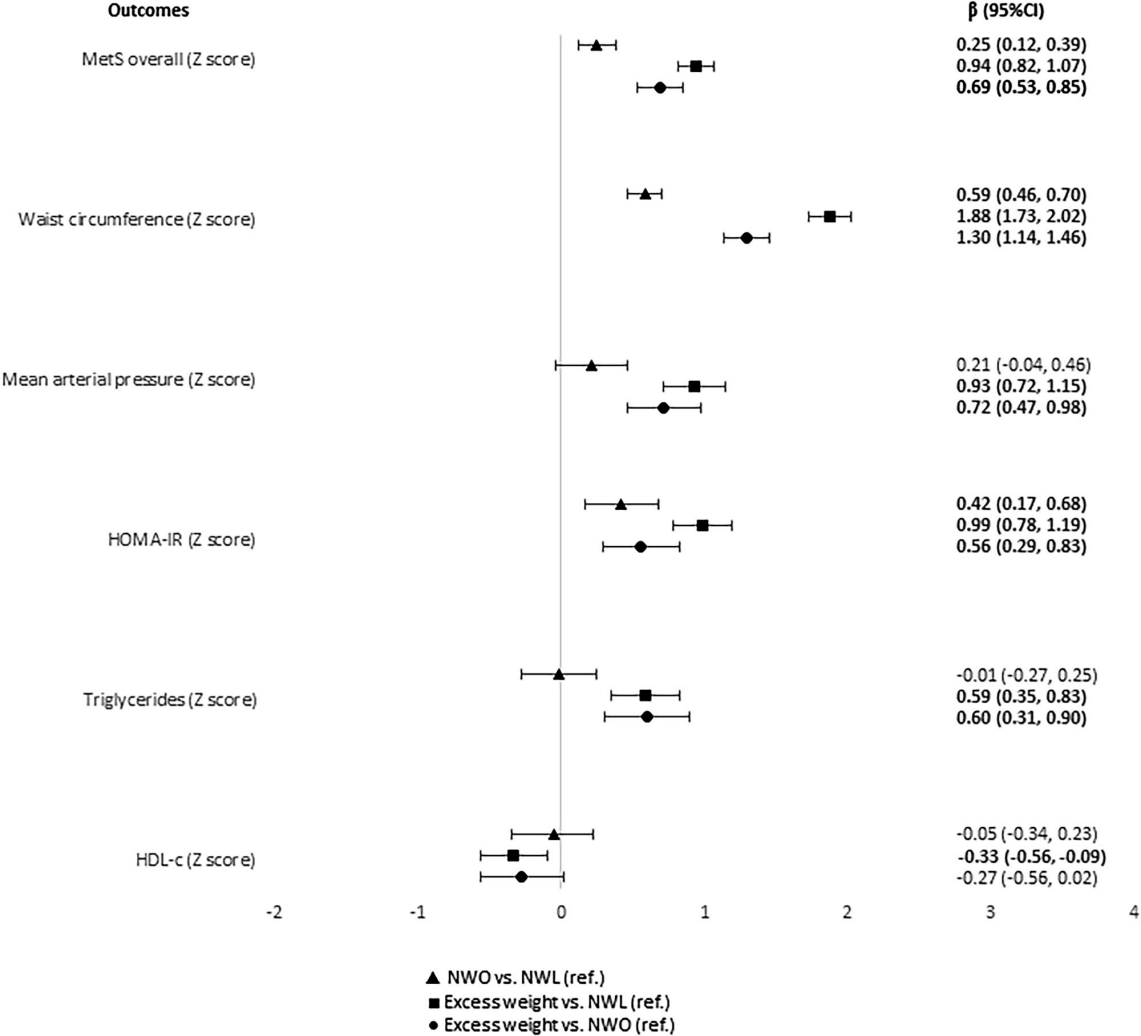
Coefficients and confidence intervals considering the relationship of obesity phenotypes (exposure) with metabolic syndrome (MetS) scores and its components (outcomes) in children. 95% CI 95% confidence interval, MetS metabolic syndrome, HOMA-IR homeostasis model assessment for insulin resistance, HDLc high-density lipoprotein cholesterol, NWO normal-weight obesity, NWL normal-weight lean, Ref reference. From multiple linear regression adjusted by per capita income, screen time and caloric intake. NWL normal-weight and adequate body fat. NWO normal-weight and high body fat. Excess weight: overweight/obesity and high body fat.

**Fig. 2:**
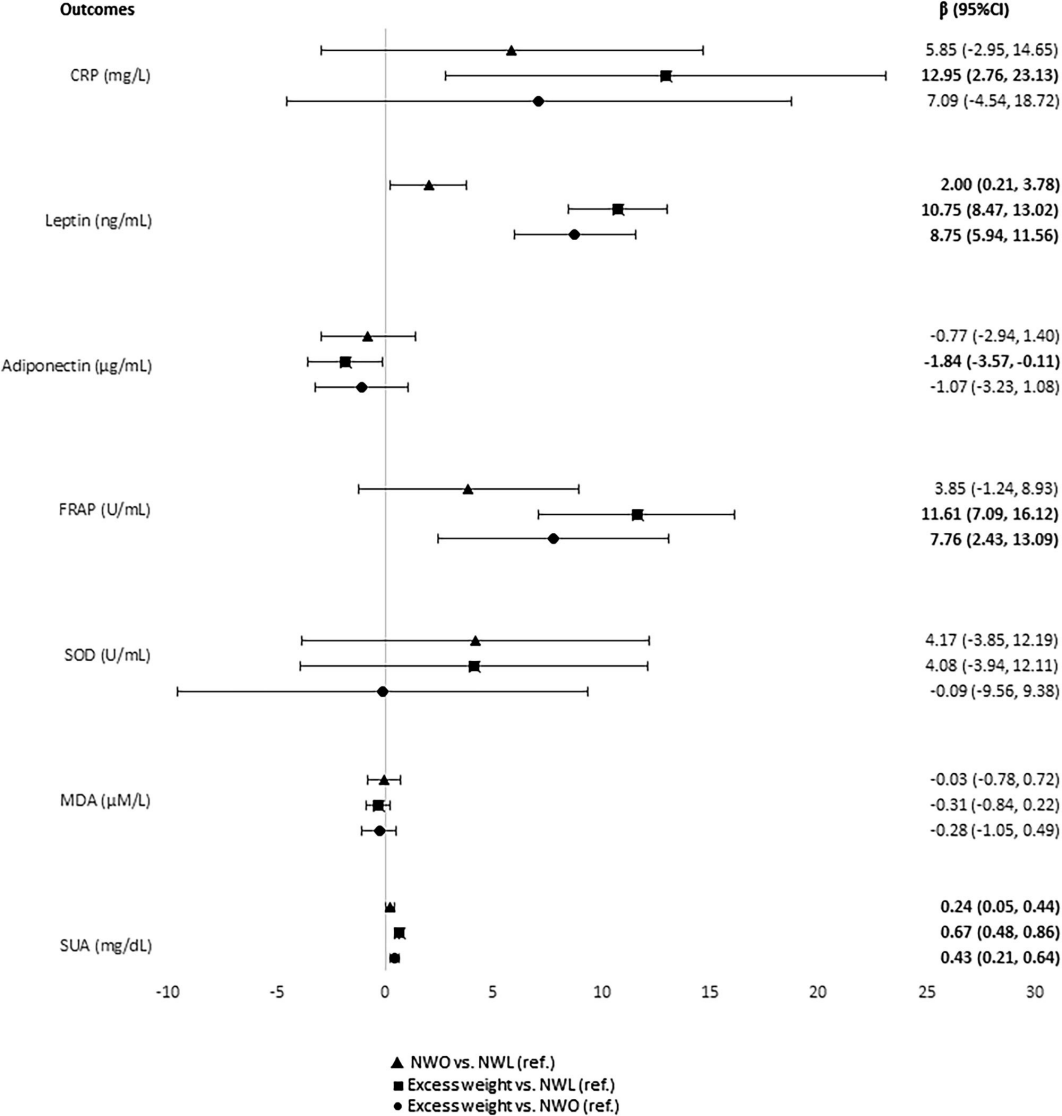
Coefficients and confidence intervals considering the relationship of obesity phenotypes (exposure) with markers of inflammation, and anti- and oxidative status (outcomes) in children. 95% CI 95% confidence interval, CRP C-reactive protein, FRAP ferric reducing antioxidant power, SOD superoxide dismutase, MDA malondialdehyde, SUA serum uric acid. NWO normal-weight obesity, NWL normal-weight lean, Ref reference. From multiple linear regression adjusted by per capita income, screen time, sex, age, and caloric intake. NWL normal-weight and adequate body fat. NWO normal-weight and high body fat. Excess weight: overweight/obesity and high body fat.

Among normal-weight children, we found five cardiometabolic risk factors [MetS overall (*β* = 0.25; 95% CI = 0.12, 0.39), WC (*β* = 0.59; 95% CI = 0.46, 0.70), HOMA-IR (*β* = 0.42; 95% CI = 0.17, 0.68), leptin (*β* = 2.00; 95% CI = 0.21, 3.78), and SUA (*β* = 0.24; 95% CI = 0.05, 0.44)] associated with the NWO phenotype, compared to NWL (Figs. 1 and 2).

#### Excess weight vs. NWL

As expected, we found a higher number (11) of cardiometabolic risk factors [MetS overall (*β* = 0.94; 95% CI = 0.82, 1.07) and all five components’ scores (WC (*β* = 1.88; 95% CI = 1.73, 2.02), Mean arterial pressure–MAP (*β* = 0.93; 95% CI = 0.72, 1.15), HOMA-IR (*β* = 0.99; 95% CI = 0.78, 1.19), triglycerides (*β* = 0.59; 95% CI = 0.35, 0.83) and HDL-c (*β* = –0.33; 95% CI = –0.56, –0.09), CRP (*β* = 12.95; 95% CI = 2.76, 23.13), leptin (*β* = 10.75; 95% CI = 8.47, 13.02), adiponectin (*β* = –1.84; 95% CI = –3.57, –0.11), FRAP (*β* = 11.61; 95% CI = 7.09, 16.12), and SUA (*β* = 0.67; 95% CI = 0.48, 0.86)] associated with excess weight, when compared to children with NWL phenotype (Figs. 1 and 2).

#### Excess body fat (excess weight vs. NWO)

Among the groups with excess body fat, we observed that eight cardiometabolic risk factors [MetS overall (*β* = 0.69; 95% CI = 0.53, 0.85), WC (*β* = 1.30; 95% CI = 1.14, 1.46), MAP (*β* = 0.72; 95% CI = 0.47, 0.98), HOMA-IR (*β* = 0.56; 95% CI = 0.29, 0.83), TG (*β* = 0.60; 95% CI = 0.31, 0.90), leptin (*β* = 8.75; 95% CI = 5.94, 11.56), FRAP (*β* = 7.76; 95% CI = 2.43, 13.09), and SUA (*β* = 0.43; 95% CI = 0.21, 0.64)] were associated with excess weight children, compared to NWO phenotype (Figs. 1 and 2).

**Fig. 3:**
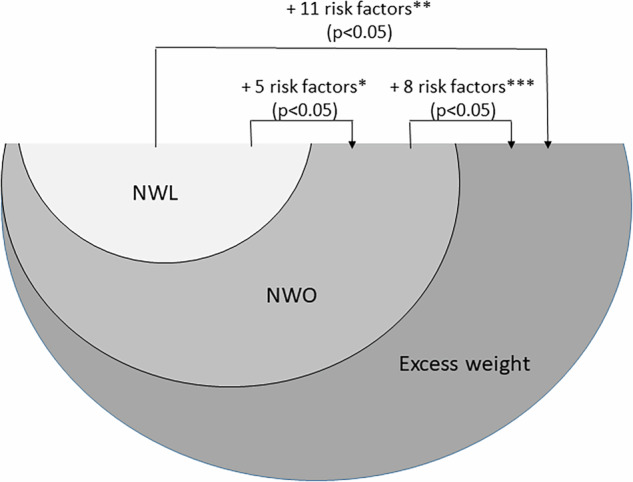
Progression of cardiometabolic risk factors according to obesity phenotypes in children. *MetS, WC, HOMA-IR, leptin, and SUA. **MetS, WC, MAP, HOMA-IR, TG, ↓HDL, CRP, leptin, ↓adiponectin, FRAP, and SUA. ***MetS, WC, MAP, HOMA-IR, TG, leptin, FRAP, and SUA. NWL normal-weight lean, NWO normal-weight obesity, MetS metabolic syndrome, WC waist circumference, MAP mean arterial pressure, HOMA-IR homeostasis model assessment for insulin resistance, TG triglycerides, HDL-c high-density lipoprotein cholesterol, CRP C-reactive protein, FRAP ferric reducing antioxidant power, SUA serum uric acid. NWL normal-weight and adequate body fat, NWO normal-weight and high body fat, Excess weight: overweight/obesity and high body fat.

Moreover, we observed a progressive increase in cardiometabolic risk factors (MetS and components scores, inflammatory markers, and anti- and oxidative markers) according to the obesity phenotypes (p trend <0.05), except for SOD and MDA (Supplementary material). The Fig. 3 summarizes the results found.

## Discussion

We observed an intermediate number of cardiometabolic risk factors in children with NWO phenotype when comparing the status of NWL to excess weight. As expected, excess weight children have a worse cardiometabolic profile. However, this study reinforces that the investigation of cardiometabolic disorders in early ages is important, independent of normal-weight by BMI.

The relationship between obesity and cardiometabolic risk factors is well established, but it is important to recognize that obesity is not a homogeneous phenomenon, and there can be different implications depending on the phenotype [33]. In this context, it is important to point out that the NWO phenotype can be a misleading and underdiagnosed condition, since people have a normal BMI, despite the excess of body fat. In our study, a significant percentage (27.3%) of normal-weight children had excess body fat, and this can increase the risk of cardiovascular diseases, type 2 diabetes, and other metabolic disorders [18, 34].

In this study, we observed a progression of cardiometabolic risk factors according to obesity phenotypes (Fig. 3). We did not find studies that investigated the NWO phenotype as an intermediate cardiometabolic risk between NWL and excess weight in the pediatric population. The progression of cardiometabolic risk factors according to obesity phenotypes can be influenced by several factors, including the percentage and distribution of body fat. Adiposity is associated with the MetS components, even in individuals with normal-weight by BMI [35]. This can be explained by the accumulation of visceral adiposity that increases the likelihood of cardiometabolic disorders [36]. The increase in BMI is also closely related to this progression, representing an important indicator of cardiovascular health [37]. This is because excessive weight gain is associated with a greater accumulation of adipose tissue, which can lead to an imbalance in the production of adipokines and hormones secreted by fat cells, which play key roles in regulating metabolism [7, 38]. In this context, there is an increase in the synthesis and secretion of leptin, as well as resistance to this adipokine, making it more pro-inflammatory, as it is involved in the production of adhesion molecules and oxidative stress in endothelial cells [39]; and CRP, a marker of subclinical inflammation [4]. In addition, adiposity and the presence of cardiometabolic disorders have been inversely related to adiponectin [40, 41], as well as being associated with increased oxidative stress [8].

Despite FRAP being an antioxidant marker, we found that children with excess weight presented higher levels of this marker than those of normal-weight (NWL and NWO). This association between obesity and FRAP can be explained by the high intake of ultra-processed foods (UPF). FRAP is a food-influenced marker [42], and UPFs have antioxidant compounds as food preservatives [43]. Thus, a high intake of UPF may contribute to an increase in this marker in children with obesity. Contrary to our hypothesis, we found no association between the oxidative stress markers SOD (an antioxidant marker) and MDA (a pro-oxidant marker) with obesity phenotypes. According to adiposity, levels of reactive oxygen species increase and exceed the capacity of the antioxidant system [44, 45]; however, the relationship between metabolic disorders and oxidative stress is still controversial in the child population, and continues to be a subject of debate. Previous studies have shown a positive association of MDA [5, 46] and a positive [5, 47] and negative [46] association of SOD, with obesity in children. We emphasize the need for more studies investigating this relationship in children, especially in the public with the NWO phenotype.

Excess SUA has been linked to oxidative stress, inflammation, insulin resistance and obesity, as well as other metabolic changes [11, 48]. However, the associations of SUA with childhood obesity phenotypes have not been fully elucidated in the literature. In our study, we found that excess fat is an aggravating factor for the increase in this metabolite, even in children of normal weight. Corroborating our findings, previous studies carried out on adults have shown an association between SUA and body adiposity, including in adults with normal-weight [49, 50] and that a higher quartile of body fat percentage was associated with higher levels of SUA [49].

It is important to point out that although BMI is considered a useful tool for obesity diagnosis, evidence suggests that a significant percentage of children may be at risk of being misdiagnosed as healthy if obesity is defined solely on the basis of this index [17]. In this sense, current BMI-based measures of obesity may hinder clinical approaches to health care and policy. Our study showed that children with normal weight and a high percentage of body fat had a significantly higher cardiometabolic risk compared to those with normal weight and no excess fat. These results reinforce the problem of underreporting of excess adiposity and the potential hidden cardiometabolic risk in children with normal weight by BMI/age. Therefore, the inclusion of adiposity measurements, such as body fat percentage and waist circumference, is important in clinical practice [35, 51, 52]. We particularly highlight the use of WC, considering that (1) it is the most accessible to nutritionists and pediatricians in health services; (2) this measure has already been considered vital for assessing or adequately managing adverse health risks [53, 54]; (3) our results demonstrated the importance of this measure to evaluate cardiometabolic risk in the pediatric population, regardless of BMI, as other studies [19, 55]. Our findings also reinforce the importance of evaluation of cardiometabolic risk in children, mainly considering the MetS and components scores (WC, HOMA, MAP, and TG) and SUA in attending public health, once they presented lower cost, in comparison to leptin, adiponectin, and FRAP.

We have several strengths of this study. To our knowledge, this is the first study with Brazilian children that compared the different obesity phenotypes with subclinical inflammation and levels of FRAP and SUA. It has a representative sample of school children, and the body fat was assessed by DXA, a reference method for assessing body composition. In addition, we used a set of adjustment variables, including demographic, economic and lifestyle variables, in a broader way when compared to the literature [19, 32, 56, 57]. However, as a limitation, the possibility of residual confounding caused by genetic factors, other socioeconomic indicators and sexual maturation cannot be ruled out. Moreover, the temporality relation cannot be established in this investigation.

In conclusion, an intermediate number of cardiometabolic risk factors was observed in children with the NWO phenotype, when compared with the NWL to excess weight status. We reinforce that the evaluation of cardiometabolic risk factors is necessary in normal-weight children, independent of BMI, since the NWO phenotype may be overlooked in children attending health services. Therefore, the WC, blood pressure, SUA, and lipid profile are important tools in clinical practice, even in children of normal weight, once they are cheap and easy to implement in health services.

## Supplementary information

Supplementary material

## Data Availability

The datasets are not publicly available due to confidentiality and controlled access policies. Anonymized data may be obtained from the corresponding author upon reasonable request.
