# Supplementary material for: Progressive increase of cardiometabolic risk in Brazilian children according to obesity phenotypes
Source: Eur J Clin Nutr. 2026 Feb 5;80(3):270–7. doi: 10.1038/s41430-026-01700-x (PMC13008770; doi:10.1038/s41430-026-01700-x)
Supplement: Supplementary file 1 — Supplementary material [file 41430_2026_1700_MOESM1_ESM.docx]

Supplementary table 1. Coefficients and confidence intervals considering the relationship of obesity phenotypes (exposure) with metabolic syndrome (MetS) and components scores, markers of inflammation, anti- and oxidative status (outcomes) in children. Viçosa, Minas Gerais, Brazil, 2015

| Outcomes | Children | | | | | p trend^*^ |
| --- | --- | --- | --- | --- | --- | --- |
|  | NWL | NWO | | Excess weight | |  |
|  |  | Unadjusted difference  Coef. (95%CI) | Adjusted difference^*^  Coef. (95% CI) | Unadjusted difference  Coef. (95%CI) | Adjusted difference^*^  Coef. (95% CI) |  |
| *MetS and components scores* |  |  |  |  |  |  |
| MetS overall (Z score) | Ref. | **0.24 (0.10, 0.38)** | **0.25 (0.12, 0.39)** | **0.94 (0.81, 1.06)** | **0.94 (0.82, 1.07)** | **<0.001** |
| Waist circumference (Z score) | Ref. | **0.57 (0.45, 0.68)** | **0.59 (0.46, 0.70)** | **1.87 (1.73, 2.01)** | **1.88 (1.73, 2.02)** | **<0.001** |
| Mean arterial pressure (Z score) | Ref. | 0.16 (-0.08, 0.41) | 0.21 (-0.04, 0.46) | **0.91 (0.69, 1.12)** | **0.93 (0.72, 1.15)** | **<0.001** |
| HOMA-IR (Z score) | Ref. | **0.40 (0.14, 0.65)** | **0.42 (0.17, 0.68)** | **0.97 (0.76, 1.17)** | **0.99 (0.78, 1.19)** | **<0.001** |
| Triglycerides (Z score) | Ref. | 0.02 (-0.24, 0.27) | -0.01 (-0.27, 0.25) | **0.61 (0.38, 0.85)** | **0.59 (0.35, 0.83)** | **<0.001** |
| HDL-c (Z score) | Ref. | -0.02 (-0.31, 0.27) | -0.05 (-0.34, 0.23) | **-0.31 (-0.53, -0.08)** | **-0.33 (-0.56, -0.09)** | **0.007** |
| *Inflammatory markers* |  |  |  |  |  |  |
| CRP (mg/L) | Ref. | 4.03 (-4.22, 12.28) | 5.85 (-2.95, 14.65) | **11.77 (2.26, 21.29)** | **12.95 (2.76, 23.13)** | **0.012** |
| Leptin (ng/mL) | Ref. | **2.30 (0.66, 3.95)** | **2.00 (0.21, 3.78)** | **11.00 (8.66, 13.34)** | **10.75 (8.47, 13.02)** | **<0.001** |
| Adiponectin (µg/mL) | Ref. | -0.51 (-2.62, 1.60) | -0.77 (-2.94, 1.40) | **-1.71 (-3.40, -0.02)** | **-1.84 (-3.57, -0.11)** | **0.038** |
| *Anti- and oxidative markers* |  |  |  |  |  |  |
| FRAP (U/mL) | Ref. | 2.85 (-2.27, 7.97) | 3.85 (-1.24, 8.93) | **10.91 (6.48, 15.33)** | **11.61 (7.09, 16.12)** | **<0.001** |
| SOD (U/mL) | Ref. | 5.33 (-2.99, 13.66) | 4.17 (-3.85, 12.19) | 4.95 (-3.17, 13.06) | 4.08 (-3.94, 12.11) | 0.302 |
| MDA (µM/L) | Ref. | -0.30 (-1.07, 0.47) | -0.03 (-0.78, 0.72) | -0.47 (-1.01, 0.06) | -0.31 (-0.84, 0.22) | 0.255 |
| Serum uric acid (mg/dL) | Ref. | **0.27 (0.08, 0.46)** | **0.24 (0.05, 0.44)** | **0.69 (0.51, 0.88)** | **0.67 (0.48, 0.86)** | **<0.001** |

NWL: normal-weight lean. NWO: normal-weight obesity. Coef.: coefficient. 95% CI: 95% confidence interval. Ref.: reference. HOMA-IR: homeostasis model assessment for insulin resistance. HDL-c: high-density lipoprotein cholesterol. CRP: C-Reactive Protein. FRAP: ferric reducing antioxidant power. MDA: malondialdehyde. SOD: superoxide dismutase.

From linear regression (Values in bold: p < 0.05). ^*^Adjusted by per capita income, screen time, sex, age, and caloric intake.

Supplementary table 2. Metabolic syndrome (MetS) and components scores, markers of inflammation, and anti- and oxidative status in children with excess body fat (excess weight vs. NWO phenotype). Viçosa, Minas Gerais, Brazil, 2015

| Outcomes | NWO | Excess weight | |
| --- | --- | --- | --- |
|  |  | Unadjusted difference  Coef. (95%CI) | Adjusted difference ^*^  Coef. (95% CI) |
| *MetS and components scores* |  |  |  |
| MetS overall (Z score) | Ref. | **0.70 (0.53, 0.86)** | **0.69 (0.53, 0.85)** |
| Waist circumference (Z score) | Ref. | **1.30 (1.14, 1.47)** | **1.30 (1.14, 1.46)** |
| Mean arterial pressure (Z score) | Ref. | **0.75 (0.49, 1.00)** | **0.72 (0.47,** **0.98)** |
| HOMA-IR (Z score) | Ref. | **0.57 (0.30, 0.84)** | **0.56 (0.29, 0.83)** |
| Triglycerides (Z score) | Ref. | **0.60 (0.30, 0.89)** | **0.60 (0.31, 0.90)** |
| HDL-c (Z score) | Ref. | -0.29 (-0.58, 0.01) | -0.27 (-0.56, 0.02) |
| *Inflammatory markers* |  |  |  |
| CRP (mg/L) | Ref. | 7.74 (-4.01, 19.50) | 7.09 (-4.54, 18.72) |
| Leptin (ng/mL) | Ref. | **8.70 (5.86, 11.53)** | **8.75 (5.94, 11.56)** |
| Adiponectin (µg/mL) | Ref. | -1.20 (-3.34, 0.94) | -1.07 (-3.23, 1.08) |
| *Anti- and oxidative markers* |  |  |  |
| FRAP (U/mL) | Ref. | **8.06 (2.62, 13.49)** | **7.76 (2.43, 13.09)** |
| SOD (U/mL) | Ref. | -0.39 (-10.29, 9.52) | -0.09 (-9.56, 9.38) |
| MDA (µM/L) | Ref. | -0.17 (-0.98, 0.64) | -0.28 (-1.05, 0.49) |
| Serum uric acid (mg/dL) | Ref. | **0.42 (0.21, 0.63)** | **0.43 (0.21, 0.64)** |

NWO: normal-weight obesity. Coef.: coefficient. 95% CI: 95% confidence interval. Ref.: reference. HOMA-IR: homeostasis model assessment for insulin resistance. HDL-c: high-density lipoprotein cholesterol. CRP: C-Reactive Protein. FRAP: ferric reducing antioxidant power. MDA: malondialdehyde. SOD: superoxide dismutase.

From linear regression (Values in bold: p < 0.05). ^*^ Adjusted by per capita income, screen time, sex, age, and caloric intake.
